# Development of Binaural Sensitivity: Eye Gaze as a Measure of Real-time Processing

**DOI:** 10.3389/fnsys.2020.00039

**Published:** 2020-07-07

**Authors:** Z. Ellen Peng, Alan Kan, Ruth Y. Litovsky

**Affiliations:** ^1^Waisman Center, University of Wisconsin-Madison, Madison, WI, United States; ^2^School of Engineering, Macquarie University, Sydney, NSW, Australia

**Keywords:** binaural hearing, interaural time difference, development, eye gaze, auditory processing

## Abstract

Children localize sounds using binaural cues when navigating everyday auditory environments. While sensitivity to binaural cues reaches maturity by 8–10 years of age, large individual variability has been observed in the just-noticeable-difference (JND) thresholds for interaural time difference (ITD) among children in this age range. To understand the development of binaural sensitivity beyond JND thresholds, the “looking-while-listening” paradigm was adapted in this study to reveal the real-time decision-making behavior during ITD processing. Children ages 8–14 years with normal hearing (NH) and a group of young NH adults were tested. This novel paradigm combined eye gaze tracking with behavioral psychoacoustics to estimate ITD JNDs in a two-alternative forced-choice discrimination task. Results from simultaneous eye gaze recordings during ITD processing suggested that children had adult-like ITD JNDs, but they demonstrated immature decision-making strategies. While the time course of arriving at the initial fixation and final decision in providing a judgment of the ITD direction was similar, children exhibited more uncertainty than adults during decision-making. Specifically, children made more fixation changes, particularly when tested using small ITD magnitudes, between the target and non-target response options prior to finalizing a judgment. These findings suggest that, while children may exhibit adult-like sensitivity to ITDs, their eye gaze behavior reveals that the processing of this binaural cue is still developing through late childhood.

## Introduction

Children rely on spatial hearing skills, such as being able to locate sound sources around them, to successfully navigate in everyday environments. By 4–5 years of age, typically developing children show adult-like performance on a range of spatial hearing abilities, including spatial acuity and localization accuracy (Deun et al., 2009; Grieco-Calub and Litovsky, 2010; Zheng et al., 2015). Maturation of spatial hearing abilities also promotes speech understanding in noise, whereby spatial cues are involved in enabling segregation of target speech from background noise (Litovsky, 2005; Garadat and Litovsky, 2007; Vaillancourt et al., 2008; Murphy et al., 2011; Misurelli and Litovsky, 2015). Similar to other auditory abilities (Lutfi et al., 2003), children also demonstrate large individual variability in spatial hearing abilities, indicating that unique individual developmental trajectories are unfolding.

The developmental trajectory of spatial hearing in children with normal hearing (NH) has been studied in the free field, whereby sound localization accuracy is often quantified with root-mean-squared (RMS) errors. For adult listeners, RMS errors for localizing sounds on the horizontal plane are typically between 2° and 10° (for a review see Middlebrooks and Green, 1991). For the best-performing children, by 4–5 years of age, RMS errors are within the range observed in adults. However, there is a range of individual differences as reported across different studies; between 6.5° and 20.8° (Zheng et al., 2015), 8.9° and 29.2° (Grieco-Calub and Litovsky, 2010), and on average about 6°–10° (Deun et al., 2009). Even children as young as 2–3 years can show RMS errors as low as 11.2° on average (Bennett and Litovsky, 2019). Another aspect of spatial hearing is localization acuity, which has been measured with the minimum audible angle (MAA), the smallest angular change in source location that is reliably discriminated. MAA is 4°–6° in 18-month-olds and becomes more adult-like (1°–2°) by 5 years of age (Litovsky, 1997).

When sounds reach the ears from source locations presented in free field, acoustic cues in the form of interaural differences, known as binaural cues, are available for spatial hearing. These include interaural time differences (ITDs) in the temporal fine structure of the signal at low frequencies, below approximately 1,500 Hz. For sounds above 1,500 Hz, binaural cues include interaural level differences (ILDs) and ITDs in the envelope of modulated signals (Middlebrooks and Green, 1991). Studies to date comparing the role of ILD and ITD in sound localization have focused on using high-frequency signals with envelope modulation, by isolating these cues and presenting sounds over headphones (e.g., Macpherson and Middlebrooks, 2002). Sensitivity to these cues has been quantified using a just-noticeable-difference (JND) threshold, which measures the smallest detectable change in the magnitude of a binaural cue. For NH adult listeners, the best envelope ITD sensitivity is captured by using transposed tones centered at a high frequency (e.g., 4 kHz) with a low-frequency envelope modulation rate [e.g., 128 Hz; (Bernstein and Trahiotis, 2002)]. A recent study compared NH children’s JND thresholds for stimuli with transposed tone vs. Gaussian envelope tone (GET). Their findings suggest that, by 8–10 years of age, JNDs for envelope ITDs and ILDs are not significantly different between children and adults (Ehlers et al., 2016). In addition, when tested in a lateralization task, i.e., the ability to map the intracranial positions of stimuli with varying ITD or ILD, 8- to 10-year-old children showed similar performance to that observed in adults (Ehlers et al., 2016). While JNDs with envelope ITDs and ILDs demonstrate adult-like performance on the group level, NH children exhibited large individual differences. In addition, their behavior was generally more indecisive during decision-making, which was not captured in the perceptual response during testing. Studies on other auditory skills show that, into late childhood and even adolescence, children typically exhibit individual variability that is much greater than that among adults (Lutfi et al., 2003; Moore et al., 2011), but very little is known about the underlying mechanisms involved in binaural decision-making processes in children.

This study focused on expanding our understanding of individual variability in binaural cue processing. We drew inspiration from the classic “looking-while-listening” paradigm (for an overview see Fernald et al., 2008), which transformed the field of language perception to study spoken language processing in very young children (for examples see Saffran et al., 1996; McMurray and Aslin, 2004; Grieco-Calub and Litovsky, 2010). This paradigm typically provides a time series of looking behavior that can be analyzed to infer decision-making processing during a perceptual task. In a recent study by Winn et al. (2019), the anticipatory eye gaze paradigm was modified to study processing strategies of ITD and ILD cues in a left–right discrimination task in NH adults. They found that, at supra-threshold stimulus magnitudes beyond the JND, the speed at which adults looked to the target side of the computer screen became faster as the magnitude of the binaural cue became more salient.

The use of the anticipatory eye gaze paradigm, or the slightly more complex “visual world” paradigm (VWP), has not been used to study binaural processes in children. In part, this is due to the requirement in designing an experimental task that will provide meaningful eye gaze time courses. Anticipatory eye gaze paradigms typically require forced-choice responses with a limited number of visual objects displayed to the listener on a computer screen. The participant is trained to fixate on a center location on the computer screen and to direct their eye gaze towards the target object that is consistent with the auditory signal after the sound presentation is terminated. During each trial, an eye-tracking camera continuously records the participant’s looking position on the screen to provide inferences on decision-making behaviors. For instance, from the eye gaze recordings, we could capture the time lapse from stimulus onset to when a participant looked to the response to quantify delays in decision-making. In addition, the continuous eye-gaze monitoring also provides a measure of uncertainty during decision-making, such as frequent fixation changes between response options, prior to submitting a button-press response. Together, we can discern a listener’s decision-making process when listening to binaural cues at different magnitudes.

In the current study, we combined eye gaze tracking with psychoacoustic measurement of ITD JND thresholds to reveal the decision-making time course during ITD processing, and to further understand the source of individual variability observed in ITD JND thresholds among children. While children have previously demonstrated adult-like performance in localization and MAA tasks, we hypothesized that mechanisms underlying the decision-making process using binaural cues, such as ITDs, may not have fully developed in children. This is because both sensory processing and cognition have been shown to develop into late childhood (Werner, 2012). Hence, we anticipated that children would differ from adults in that eye gaze data would reveal longer delay and higher uncertainty than adults during the decision-making process, as well as taking more time than adults before submitting a button-press response.

## Materials and Methods

### Participants

Two groups of NH children participated in the study: (1) 10 younger children (8 years, 0 months to 10 years, 9 months; *M* = 9.1 years); and (2) 10 older children (11 years, 1 month to 14 years, 10 months; *M* = 12.6 years). All children were typically developing with no known developmental delays, or hearing or speech impairments. On the day of testing, none of the children had a known illness or ear infection based on parental report. A group of 10 NH young adults (18 years to 24 years; *M* = 21.1 years) also participated in the study and performed the same tasks. All listeners had pure tone hearing thresholds at or below 25 dB hearing levels in both ears at octave band center frequencies between 250 and 8,000 Hz.

All experimental protocols and procedures were approved by the Health Sciences Institutional Review Board at the University of Wisconsin-Madison. Parents or legal guardians signed informed consents and children signed an assent form. Children were compensated $7.50/h in addition to multiple small prizes provided during the test session to keep the child attentive and motivated. All adult listeners provided written consent and received compensation of $8.00/h for the test session. All listeners completed the experiment in a single visit that lasted no longer than 2 h, which included consent, hearing screen, and frequent breaks.

### Stimuli

Stimuli consisted of 4-kHz carrier tones that were amplitude-modulated by an envelope with a frequency of 128 Hz. Stimulus duration was 300 ms, with 20 ms on- and off-ramps. The sampling frequency of the stimulus was 48 kHz. Pink noise was added to mask potential low-frequency distortion artifacts from the modulation tone at 25 dB signal-to-noise ratio. All sounds were calibrated to be presented at 65 dBA. The choice of the carrier and envelope was based on prior work by Bernstein and Trahiotis (2002) to maximize access to envelope ITD in listeners with NH. In each trial, three stimuli were presented with an inter-stimulus interval of 300 ms. The first and second intervals always contained a 0-μs ITD, and the envelope ITD in the third interval was one of the test magnitudes. In this article, positive ITDs indicated right-ward direction with leading acoustic signals in the right ear and were achieved by time-delaying the left ear signals by the target ITD, and vice versa for negative ITDs indicating left-ward direction. To allow enough repetitions in the eye gaze data for each stimulus magnitude, the method of constant stimuli was used in this experiment to estimate the ITD JND at 70.7% correct. A set of ITDs of 20, 80, 140, 200, and 400 μs was chosen based on pilot data to best capture the JNDs at 70.7% accuracy among both adult and child listeners. The ILD was set to 0 dB in all stimuli. All listeners completed 30 repetitions for each ITD magnitude, where half of the trials contained left-leading ITDs and the other contained half right-leading ITDs. The total trials were divided into blocks of 30–50 trials depending on the child’s progress, where ITD magnitudes were randomized in their order of presentation within the block. For any children who performed consistently at chance on trials with smaller ITDs during the first half of the testing session, the 20-μs ITD condition was replaced by 700-μs ITD, which is the largest possible ITD from a standard manikin head at 4 kHz (Kuhn, 1977). The substitution allowed a better estimation of performance for children who were not sensitive to the smaller ITDs.

### Experiment Setup and Testing Procedure

Participants were tested in a double-walled sound booth (Acoustic System, TX, USA). Listeners sat at a table with a 19-inch LCD monitor (1,280 by 1,024 pixel resolution) positioned at 63 cm away. A chin rest was used to maintain the distance of the head to the monitor and restrict head movement. An EyeLink 1000 eye-tracking camera (SR Research, Kanata, ON, Canada) sat on the table to capture participant’s eye gaze movements at 1,000 Hz sampling rate. An RME Fireface sound card was used to deliver sounds to a pair of Sennheiser HD 600 circumaural headphones (Sennheiser, Hanover, Lower Saxony Germany). Audiovisual stimuli were presented using custom software written in MATLAB (Mathworks, Natick, MA, USA). The Psychophysics Toolbox (v3.0.14; Pelli, 1997) was used to maintain the synchronization of audiovisual stimulus presentation with the eye-tracking camera.

**Figure 1:**
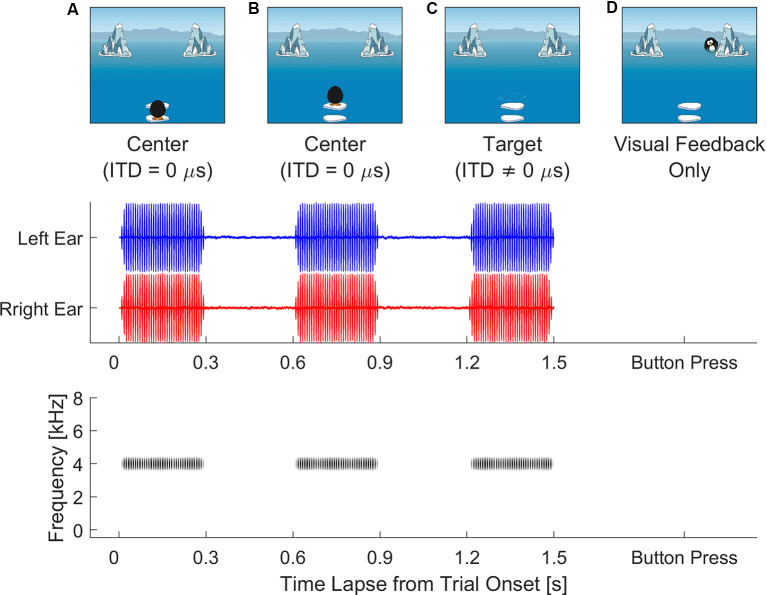
Video sequence of visual stimuli displayed on screen (top row, **A–D**) after subject initiates the trial. Audio stimuli (middle and bottom rows) are synchronized to play back two centering transposed tones [interaural time difference (ITD) = 0 μs] while the cartoon penguin is visibly hopping on screen **(A,B)**. After the cartoon penguin disappears **(C)**, the third and target tone carrying a non-zero ITD plays. An inter-stimulus interval of 300 ms is included in the playback of the transposed tones. Listeners judge the moving direction of the target tone and use a mouse to press the response button (i.e., left or right iceberg). Immediately after the button press, visual feedback **(D)** is provided as the cartoon penguin reappears from behind the correct response button, e.g., the iceberg on the right.

The experiment was conducted as an interactive video game displayed on the full screen. Figure 1 illustrates the stimulus sequence during each trial. At the beginning of each trial, listeners were instructed to visually follow the cartoon penguin, which took two jumps before taking a dive and disappearing from view. The three auditory stimuli were synchronized with cartoon penguin’s visual prompts. During each of the two intervals, the penguin jumped along the monitor midline and the auditory stimulus had an ITD of 0 μs. The penguin then took a dive into the ocean and disappeared from view, after which the third auditory interval was presented with an ITD favoring the right or left to cue the direction in which the penguin had taken. During task familiarization, listeners were told that, after the penguin took the final dive, it would swim toward one of the icebergs either on the right or left on the computer screen, the clue to which iceberg was in the last sound they hear. Listeners’ task was to find the penguin, based on the target ITD direction, by looking toward either iceberg. Once a final decision was made, they were instructed to use a computer mouse to choose either iceberg by clicking on it. To avoid visual distraction, the mouse cursor was only visible on the screen after the offset of the third stimulus interval. Immediately after the listener pressed the response button, feedback was provided with the cartoon penguin reappearing behind the iceberg that corresponded to the correct ITD direction. There was also an alternate version of the video sequence with a cartoon bunny jumping to hide behind bushes. The children were given a choice between the two versions. Both versions of the video game shared the same auditory aspects, as well as the same pixel area definition for the response buttons (i.e., icebergs or bushes) on the monitor screen to ensure equivalent eye gaze tracking results. The recording of eye gaze positions on the monitor screen, as pixels in *x*- and *y*-coordinates, always started 50 ms before the onset of the third interval and terminated when the participant clicked on the response box.

## Data Analysis

### ITD JND Threshold

Each listener’s ITD JND threshold was estimated by fitting a logistic function to the percent correct responses provided *via* the button press at the end of each trial as a function of target ITD. The psignifit MATLAB toolbox (Version 3; Fründ et al., 2011) was used to fit the logistic function to the data. Since the experiment was a two-alternative forced-choice task with a chance level at 50%, ITD JNDs were estimated as the 70.7% correct point on the logistic regression curve. To compare ITD JND thresholds between age groups, pairwise comparisons using two-tailed *t*-tests were conducted with Bonferroni correction of three comparisons.

### Response Times and Saccade Counts

For each trial, two measures of response times (RT) were calculated: (1) RT to button press; and (2) RT to first saccade. The RT to button press was calculated as the time lapse from the onset of the target stimulus to when the listener submitted a response using button press. The RT to first saccade was calculated as the time lapse from target stimulus onset to the first saccade the listener initiated away from the center region towards either the target or non-target side. RT to first saccade is akin to a reaction time measure describing the time course listeners took to arrive at the initial fixation on a response.

From the raw eye gaze data, we were also able to capture saccades when listeners changed their fixation after the initial saccade, such as from the initial fixation to the final response. When a listener completed a trial without fixation switches, the saccade count is 1. Each fixation switch between target and non-target regions following the initial fixation increased the saccade count by 1.

### Modeling Time Course of Decision-Making From Eye Gaze Data

**Figure 2:**
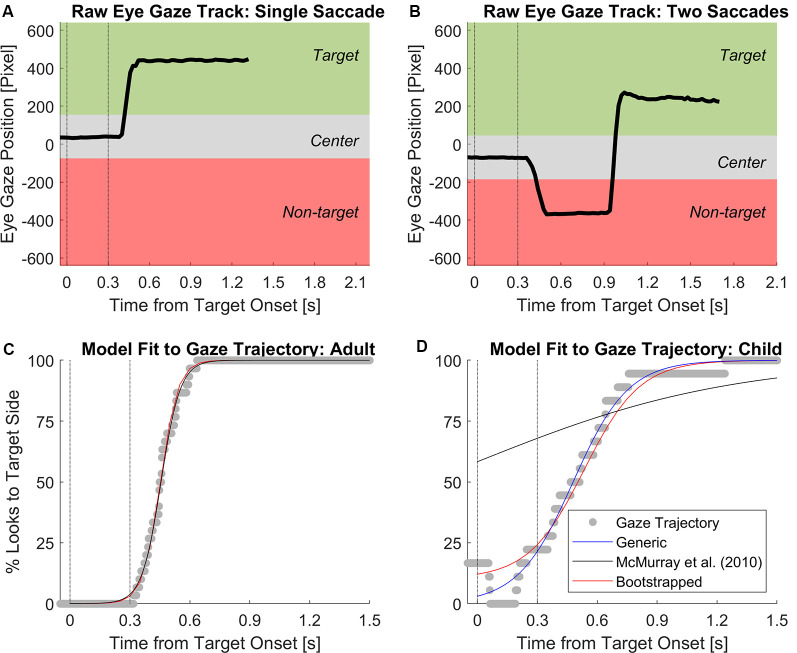
Sample time courses of eye gaze position on screen of **(A)** a trial completed with a single fixation to the target and **(B)** a trial with an initial fixation to first non-target then the target. The center region was defined as ±115 horizontal pixels from the averaged fixation position within a 100-ms window around the onset of the target interval. Example logistic regression fits to gaze trajectory from **(C)** one adult and **(D)** one child tested in 400 μs of ITD. Vertical dotted lines mark the onset and offset of the target interval. The generic logistic regression fit using $\frac{1}{1+e^{-\frac{t-\alpha }{\beta }}}$ is plotted in blue. The modified logistic regression using the equation from McMurray et al. (2010) is plotted in black; poor fitting was due to the non-zero % looks at target onset. The regression with the bootstrapping algorithm from Wichmann and Hill (2001) is shown in red, which had the best model fits on individual levels among all models.

Eye gaze data were analyzed separately from JND thresholds. Figures 2A,B illustrate sample time courses of eye gaze position on the computer screen. For analysis of eye gaze data, the gaze positions on screen were coded based on their three vertical regions defined as the center, target side, and non-target side. Each region spanned the full height of the screen. The center region was defined as ±115 horizontal pixels (~10% of the screen width) around the averaged fixation location within a 100-ms window around the onset of the target stimulus (i.e., 50 ms before and 50 ms after). The target side and non-target side regions were defined as the vertical screen areas outside the center region. The gaze positions within the target side region were coded with the numerical value of 1, and remaining gaze positions (non-target side and center) were coded with the numerical value of 0. Over repeated test trials of the same ITD magnitude, the individual time courses of discretized fixations were time-aligned and transformed into a single time course of percent looks towards the target side. In the present study, we defined an eye gaze trajectory as the time course of % looks to the target side for each listener per ITD magnitude. To understand the time course of ITD cue processing, we undertook careful examinations of several modeling techniques used in the literature to model the time-series data of eye gaze trajectory accurately. Three different model fits were considered: (1) a generic logistic regression fit; (2) a logistic regression with modified parameters based on modeling eye gaze trajectories in the VWP (McMurray et al., 2010); and (3) a logistic regression with bootstrapping using algorithms developed by Wichmann and Hill (2001). Both the (Wichmann and Hill, 2001) and (McMurray et al., 2010) models keep the % looks to target side unconstraint at *t* = 0 and end of the trial, allowing these two parameters to be adjusted to individual values depending on the gaze trajectory. Figures 2C,D illustrate examples of the logistic regression curve fit to individual eye gaze trajectories for one adult and one child, respectively. For adults, all three models generated excellent goodness-of-fit in *r*^2^ > 0.99. However, for children who did not always fixate at screen center (i.e., % looks > 0 at *t* = 0), the McMurray et al. (2010) model was unable to generate a good model fit (black line in Figure 2D). Overall, the Wichmann and Hill (2001) approach was best at modeling individual eye gaze trajectories for both adults and children in this study. From individual participant’s model fits using this approach, four parameters are extracted including upper and lower asymptote of the curve, as well as latency and slope estimated at 50% looks point on the curve.

### Eye Gaze Analysis Procedure

#### Data Processing

To reduce computational effort during model fitting, the eye gaze data originally measured at 1,000 Hz sampling frequency (a sample every 1 ms) was down-sampled by decimation to 20 Hz (a sample every 50 ms). From the raw eye gaze data, we observed that a single saccade typically lasted 30–50 ms. Since the dependent variable in the current analysis is the percent looks to the target side, which is an aggregated measure of all valid eye gaze tracks in the same ITD condition for each subject, the onset and offset of individual saccades are not of particular interest. Hence, any two consecutive saccades would remain in the data despite down-sampling to 20 Hz, even if participants made rapid eye gaze switches on screen.

We took careful steps in determining the validity of the eye gaze data using the following rules. In each trial, we ensured that it:

(1) contained no more than 30% blinks (i.e., missing data) in the eye gaze recording;
(2) showed agreement between the final eye gaze position and the button press;
(3) lasted <3 s or within 3 SD of trial durations from all test trials with the same ITD magnitude.

By doing so, we excluded test trials on which listeners looked to a region different from the button press or had a prolonged RT due to inattention. Linear interpolation was used to recover missing gaze positions on the screen for test trials with ≤30% blinks in the eye gaze recording. Each test trial ended when the listener submitted the button-press response; thus, trials varied in duration. To ensure all recordings ended at the same time, shorter recordings were padded at the end with the average eye gaze location from the last 10 ms of the recording. Since listeners were unlikely to submit a button press within 10 ms after initiating a saccade, extending the shorter fixation time courses using the last 10 ms retained the listener’s final gaze position prior to submitting a button press. The individual discretized fixation time courses were combined to calculate the percent of looks to the target side as a function of time. This single eye gaze trajectory was then fitted with the Wichmann and Hill’s (2001) model as described in the previous section. From the model, four parameters were extracted: (1) latency at 50% looks to the target side; (2) slope estimated at 50% looks on the curve; (3) upper asymptotes; and (4) lower asymptotes. The point of 50% looks was taken at the mid-point of the linear rise on the fitted logistic regression. In addition, the measured lower and upper asymptotes were the actual percent of looks to the target side at the stimulus onset and the end of the trial, respectively. By correlating with other behavioral measures, such as RT and saccade counts, these model parameters can be understood to relate to an individual listener’s speed of arriving at a decision (i.e., latency), uncertainty in making the decision (i.e., slope), and bias in looking behavior (i.e., lower and upper asymptotes).

### Statistical Analysis

To understand how ITD magnitude and age affect eye gaze behaviors, we used linear mixed-effects models (LMMs) with maximum likelihood estimation. LMMs allowed us to model ITD levels and age group as fixed effects, as well as individual listeners as a random effect to account for individual differences. In addition, LMMs were capable of modeling repeated measures variable with missing data, which was suitable for analyzing the effect of ITD magnitude, since some children were tested with a larger ITD level and did not share all the ITD levels tested with other listeners. The baseline model contained fixed factors of ITD magnitude and age group and individual listener as a random factor. This allowed us to examine the effect of age beyond individual differences. Using the model-comparison approach, the interaction between ITD magnitude × age group was added to the baseline model to check for significant improvement in the overall model prediction. If the addition of the interaction term was significant, a *post hoc* analysis was performed by fitting an LMM for each age group. The statistical analysis packages used in R (v3.5.1) included “lme4” (V1.1-21) for fitting LMMs.

## Results

### ITD JND Threshold and Slope of Psychometric Function

**Figure 3:**
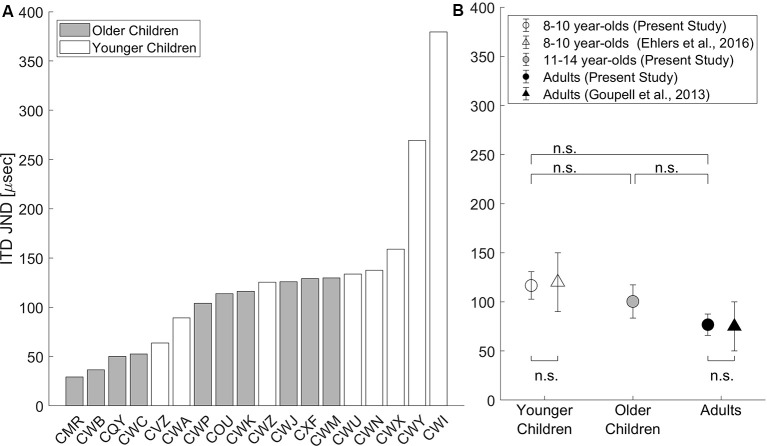
**(A)** ITD just-noticeable-differences (JNDs) estimated from button presses are shown for each child, with younger children in white bars and older children in gray bars. **(B)** Group means (±1 SE, *N* = 10 in each age group) are shown for data from the current study (circles) and previously published data (triangles; replotted with permissions). Younger children (8–10 years) are plotted in white (with CWY and CWI removed), older children (11–14 years) are plotted in gray, and adults are plotted in black. The statistical significance level was set at *α* < 0.05 with Bonferroni correction for pairwise comparisons; n.s., non-significant, *p* > 0.05.

ITD JND threshold was estimated from the button-press responses to provide a comparison of these data to that of previous psychoacoustic studies. Details of how ITD JNDs were estimated can be found in “ITD JND Threshold” section. The estimated ITD JNDs are shown in Figure 3. In the younger children group, two children who were both 8 years old had no measurable ITD JNDs and were excluded from data analysis. In Figure 3A, individual JND thresholds are plotted for all children with measurable ITD JNDs. Two children (participant ID: CWI and CWY) were identified as outliers with ITD JNDs beyond the 90th percentile and were removed from calculating the group average shown in Figure 3B.

In Figure 3B, ITD JND group mean (± standard error; SE) is shown for the younger children, older children, and adults from the present study, as well as group means (±SE) for children from Ehlers et al. (2016) and for adults from Goupell et al. (2013). On the group level, ITD JNDs replicated previous findings as confirmed by two-tailed, one-sample *t*-tests compared with the group-averaged JNDs reported for children from Ehlers et al. (2016) and for adults from Goupell et al. (2013), both *p* > 0.05. To test the age group effect, pairwise comparisons using two-tailed *t*-tests with Bonferroni corrections revealed no significant differences in ITD JNDs measured among the three age groups (all *p* > 0.05). Removal of listeners CWI and CWY did not change the outcome of the *t*-tests.

Besides ITD JND thresholds, the slope of the psychometric function at 70.7% accuracy was also extracted from the logistic regression curve fitted to the individual listener’s data. Pairwise comparisons using two-tailed *t*-tests with Bonferroni corrections suggested that the averaged slope of the ITD psychometric function was shallower in younger children (*M* = 0.20%/μs, SD = 0.11) than adults (*M* = 0.41%/μs, SD = 0.15), *t*_(16)_ = −3.41, *p* = 0.011. Averaged slope was not significantly different between older children (*M* = 0.25%/μs, SD = 0.15) vs. younger children or adults, both *p* > 0.05. By removing the two 8-year-olds with outlier ITD JNDs, the difference in JND slope was no longer significant between younger children and adults, *p* > 0.05.

### RTs and Saccade Counts

To understand the real-time processing behavior of listeners, RT to button press, RT to first saccade, and saccade counts were computed. Details of how these metrics were computed can be found in “Response Times and Saccade Counts” section. For the RT to button press, we screened the influence of including incorrect trials in the calculation of RT. By including incorrect trials, the average RT was significantly longer by 15 ms in 400 μs ITD to 40 ms in 20 μs ITD (all *p* < 0.05, two-tailed paired *t*-tests). It suggested that the decision-making processes might be different depending on the response accuracy. Hence, only correct trials were included in all subsequent analyses including eye gaze trajectories. For each listener, an average RT to button press was calculated per ITD magnitude.

#### RT to Button Press

**Figure 4:**
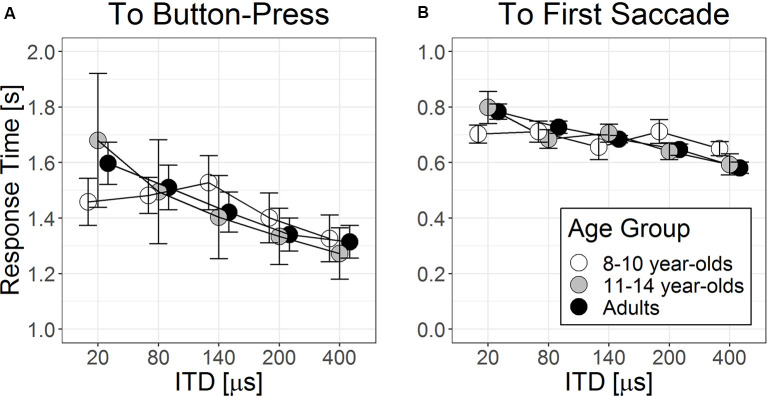
Group means (±1 SE, *N* = 10 in each age group) of response time from target stimulus onset to **(A)** button press and to **(B)** onset of the first saccade as a function of ITD magnitudes.

Figure 4A shows RT to button press as a function of ITD separately for three age groups. There was a significant main effect of ITD magnitude on RT to button press (*F*_(1,110.3)_ = 45.67, *p* < 0.001). Listeners were faster in providing correct button-press responses as ITD magnitude increased, *b* = −0.68 × 10^−4^, *t*_(110.8)_ = −5.71, *p* < 0.001. There were no significant between-group differences in RT to button press among the three age groups, *p* = 0.063 beyond individual differences ($\chi_{\left(1\right)}^{2}$ = 86.9, *p* < 0.001, as random factor). The interaction between ITD magnitude × age group did not enter as a significant predictor, $\chi_{\left(2\right)}^{2}$ = 4.56, *p* = 0.10, suggesting that the rate of reducing RT to button press with larger ITD magnitude did not differ significantly among age groups.

#### RT to First Saccade

Figure 4B shows time lapse to the first saccade when ITD magnitudes were changed for the three age groups. Similar to findings on RT to button press, the main effect of ITD magnitude on RT to first saccade was significant (*F*_(1,104.1)_ = 34.2, *p* < 0.001). Listeners were faster to arrive at the initial fixation as ITD magnitude increased, *b* = −3.99 × 10^−4^, *t*_(104.1)_ = −5.85, *p* < 0.001. Neither group effect nor its interaction with ITD magnitude was statistically significant, *p* > 0.05.

#### Saccade Counts

**Figure 5:**
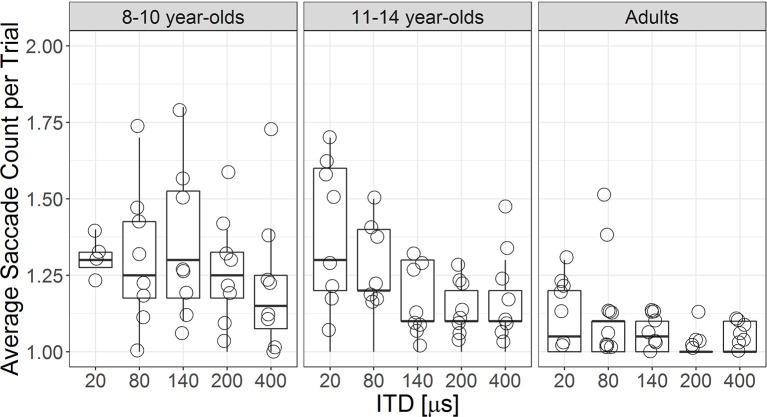
Boxplot showing individual participants’ average saccade count per trial by ITD magnitudes for each age group. The upper and lower borders of the box represent 75th and 25th percentile, respectively; whiskers represent 1.5 times above and below the interquartile range. Individual data are plotted in open symbols.

For each listener, an average saccade count per trial was calculated per ITD magnitude. Figure 5 shows the individual data of the average saccade count per trial for each ITD magnitude in each age group. Overall, children demonstrated larger variability and more saccade counts across different ITD magnitudes. LMM revealed significant main effects of ITD magnitude (*F*_(1,112.1)_ = 16.20, *p* < 0.001) and age group (*F*_(2,27.13)_ = 7.04, *p* = 0.003) beyond individual differences. Listeners made fewer saccade revisions as ITD magnitude became larger, *b* = −3.10 × 10^−4^, *t*_(112.2)_ = −4.03, *p* < 0.001. Collapsed across all ITD magnitudes, adults made fewer saccade revisions than both groups of children (vs. older children *t*_(26.9)_ = 2.71, *p* = 0.011; vs. younger children, *t*_(27.4)_ = 3.55, *p* = 0.001). Both groups of children had similar average saccade counts per trial, *p* > 0.05.

### Eye Gaze Trajectory

Listener’s eye gaze trajectories were examined in detail by fitting gaze data *via* logistic regression (see “Modeling Time Course of Decision-Making from Eye Gaze Data” section for details). This fit allows four important parameters to be extracted: lower and upper asymptotes, latency, and slope. These values provide an estimate of a listener’s bias and uncertainty when correlated with other behavioral measures.

#### Lower and Upper Asymptotes

**Figure 6:**
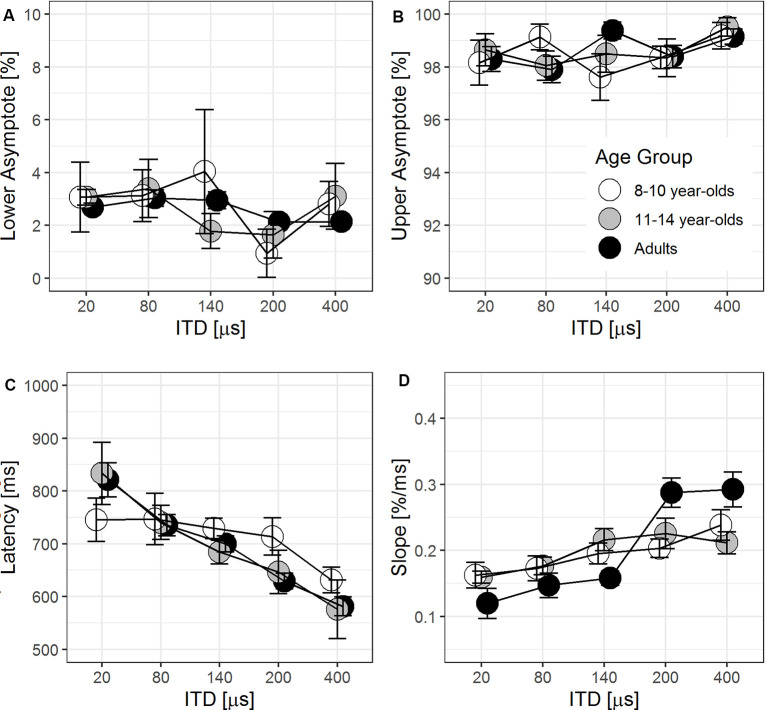
Group means (±1 SE, *N* = 10 in each age group) of parameter estimates: lower asymptote **(A)**, upper asymptote **(B)**, latency **(C)**, and slope **(D)** estimated at 50% looks to the target side from logistic regression fit to individual eye gaze trajectory.

The lower and upper asymptotes of the individual eye gaze trajectory captured the % looks to the target side estimated at the beginning and end of trials, respectively. Figures 6A,B illustrate the lower and upper asymptotes (±1 SE) as a function of ITD magnitude for three groups of listeners. To verify the logistic regression fit in capturing the asymptotes, two-tailed paired *t*-tests confirmed that there was no significant difference between the predicted value and the actual lower asymptote, *p* = 0.053, but the upper asymptote was significantly underestimated by 1.3%, *p* < 0.001. Under the inclusion criteria for individual eye gaze tracks outlined in “Data Processing" Section (section 3.4.1), the upper asymptote should approach 100% for each trajectory. Only three trajectories (~2% of data) had an upper asymptote <100%, with the smallest value at 96.1%. Such minor deviation was likely due to small jitters in maintaining the eye position on screen toward the end in a small number of test trials. Together with goodness-of-fit *r*^2^ > 0.93 for 93.5% of all individual eye gaze tracks, the logistic regression fit was very robust in modeling the eye gaze trajectory. While most adult listeners were able to consistently fixate on the screen center at the beginning of each trial, more children in both groups demonstrated a slight bias with higher estimated lower asymptote as seen with larger SE across all ITD magnitudes in Figure 6A.

#### Latency

For each tested ITD magnitude, latency is the time lapse from the onset of the target stimulus to 50% looks to the target side. In Figure 6C, the group averages of latency (±1 SE) were plotted as a function of ITD magnitudes. ITD was a significant predictor of latency, *F*_(1,109.96)_ = 67.89, *p* < 0.001. There was a significant downward trend of reduced latency as the ITD cue magnitude increased and became more salient, *b* = −0.48, *t*_(109.96)_ = −8.24, *p* < 0.001. Beyond individual differences ($\chi_{\left(1\right)}^{2}$ = 108.84, *p* < 0.001), there was no significant age group effect (*p* = 0.49), nor was there significant interaction with ITD magnitude (*p* = 0.26).

#### Slope

The slope of the eye gaze trajectory was estimated at the time point of 50% looks to the target side, where latency was also estimated. Figure 6D shows the averaged slope (±1 SE) as a function of ITD for three age groups. In the baseline LMM including only ITD magnitude and age group, slope significantly improved with increasing ITD magnitude, *F*_(1,112.4)_ = 37.57, *p* < 0.001, and varied among age groups, *F*_(2,27.1)_ = 9.39, *p* < 0.001.

The interaction between ITD magnitude and age group entered into the LMM, $\chi_{\left(2\right)}^{2}$ = 16.66, *F*_(2,111.2)_ = 8.96, *p* < 0.001. However, the main effect of age group was no longer significant (*p* = 0.25) with its interaction with ITD magnitude included in the LMM. For *post hoc* analysis on the interaction, a separate LMM including individual listener and ITD magnitude was fitted to slope in each age group. Results showed that slope improved as ITD magnitude increased for younger children, *b* = 1.23 × 10^−4^, *t*_(35.2)_ = 2.89, *p* = 0.0065, and for older children, *b* = 1.58 × 10^−4^, *t*_(37.4)_ = 2.94, *p* = 0.0056. For adults, the slope improved more drastically with larger ITD magnitudes, *b* = 4.79 × 10^−4^, *t*_(40.0)_ = 5.66, *p* < 0.001. By removing two potential outliers among adults who had a much larger slope in the 200- and 400-μs ITD conditions, the positive relationship between ITD magnitude and slope in eye gaze trajectory remained significant. The rate at which the slope increased with larger ITD magnitudes was only slightly reduced to *b* = 3.34 × 10^−4^, *t*_(32.0)_ = 4.10, *p* < 0.001, which was still more than double of that from younger and older children. For adults, the slope from eye gaze trajectory improved at a much faster rate with increasing ITD magnitude than both groups of children. Since there was no effect of age group on latency estimated at the time when the listener reached 50% looks to the target side, the steeper the slope in adults suggested that they reached the upper asymptote faster than both groups of children.

### Relationship Between Measures From Eye Gaze and Button Press

**Figure 7:**
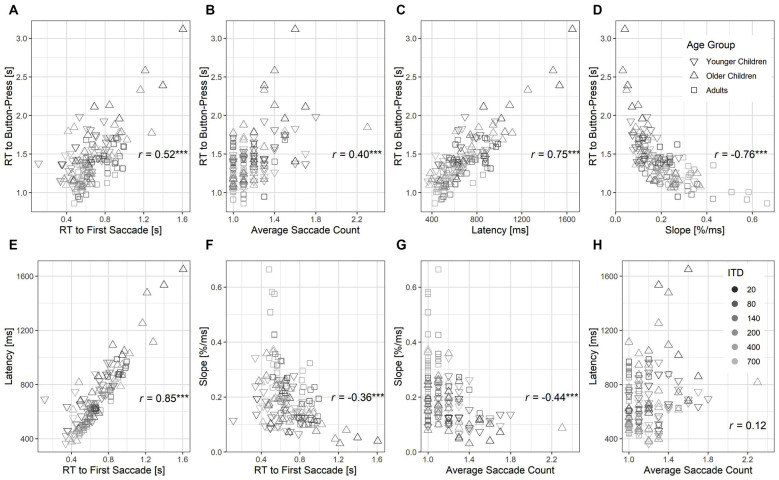
Scatter plots showing inter-relations between pairs of outcome measures from button press and eye gaze trajectory **(A–H)**. Gray scale intensity indicates ITD magnitudes. Spearman’s partial correlations calculated from *N* = 136 between each pair of measures are shown by controlling for ITD magnitudes. Significant correlations are highlighted with asterisks, ***p < 0.001.

To understand the inter-relationship between behavioral measures obtained from button press and eye gaze recordings, we correlated pairs of outcome measures using Spearman’s Rank partial correlation by controlling for ITD magnitude (see Figure 7). In comparison with RT to first saccade or average saccade count (Figures 7A,B), RT to button press was more strongly correlated with latency and slope estimated from the eye gaze trajectory (Figures 7C,D). It suggested that the latency and slope estimates were better at explaining the variance observed in RT to button press as metrics for decision-making. Furthermore, while RT to the first saccade significantly correlated with both latency and slope (Figures 7E,F), average saccade count only correlated significantly with slope (Figure 7G) but not latency (*p* > 0.05, Figure 7H). This suggests that the latency estimate was a proxy for the time lapse to first saccade, whereas the slope estimate was collectively influenced by both the time to first saccade and later fixation switches.

## Discussion

This study is the first to investigate the decision-making time course in children in the domain of binaural hearing. We used a modified “looking-while-listening” paradigm, which is ubiquitous in studies on language processing in very young children (for examples see Saffran et al., 1996; McMurray and Aslin, 2004; Grieco-Calub and Litovsky, 2010). In these studies, eye-tracking methods provide insight into understanding how stimulus features impact decision-making time course while information is being processed. We studied children with NH ages 8–14 years to test the hypothesis that age-related differences in ITD processing that are not apparent with JND threshold estimates are revealed with eye gaze measures.

Our estimates of ITD JND thresholds from button-press responses replicated the findings of Ehlers et al. (2016), that children demonstrate adult-like ITD sensitivity by 8–10 years of age. This study extends previous findings by investigating age-related differences in the decision-making time course and uncertainty when children perform an ITD discrimination task. To capture an individual’s real-time processing behavior, we evaluated several metrics that quantified the decision-making time course leading up to a behavioral response. We derived three metrics to describe the delay during the decision-making time course. First, RT to button press using a computer mouse captured the end of the decision-making time course and was used here while acknowledging that there may be potential confounds due to other developmental immaturities in this age range, such as fine motor skills (Piper, 2011). Second, time to the first saccade was a direct measure of the time listeners took to arrive at an initial fixation on a response button, revealing the earlier stage of decision-making. Third, as another window into the time course, we estimated latency from stimulus onset to the mid-point of the eye gaze trajectory. All three measures suggested that increasing ITD magnitudes resulted in faster decisions for both children and adults, corroborating findings in NH adults (Winn et al., 2019). In addition, when compared to adults, children did not take any longer to arrive at the initial fixation or the final decision, a finding that does not support our initial prediction regarding the maturation of ITD processing.

We did, however, observe an age difference in the eye gaze data that was linked to uncertainty in decision-making; children made more fixation switches between the target and non-target sides than adults across all ITD magnitudes tested. Even though the analysis only included trials with correct responses, children showed more frequent fixation switches between response options when processing binaural information prior to submitting a correct button-press response. Interestingly, uncertainty behavior was not exclusive to children. When ITD magnitude was small (e.g., 20 μs), adults also demonstrated more frequent fixation switches than when tested in larger ITD magnitudes. In the model fit to individual eye gaze trajectories, more frequent fixation switches led to shallower slopes, implying poorer processing efficiency. The slope of individual eye gaze trajectories was sensitive in capturing the age-related difference in processing ITD cues, as seen in the significant interaction between age group and ITD magnitude (Figure 6D). This finding confirmed the hypothesis that, when processing ITD, children showed greater uncertainty in decision-making than adults.

In fact, our findings from eye gaze behavior support previous reports that the development of auditory processing is likely related to cognitive factors (Lutfi et al., 2003; Buss et al., 2009; Jones et al., 2015). In auditory tasks that involve signal detection in noise, internal noise in the form of variability in the neural representation of the auditory input has been attributed to individual differences in behavioral outcomes (Buss et al., 2006). Until late adolescence, children demonstrate elevated internal noise as compared to adults in detecting tone in noise (Buss et al., 2006) and amplitude modulation (Cabrera et al., 2019). In tasks that involve cross-frequency masking, immature selective attention is also found to be responsible for poorer performances in children as compared to adults (Lutfi et al., 2003; Leibold and Neff, 2007, 2011; Jones et al., 2015). Even though frequency resolution is mature before the end of infancy (Werner, 2012), it takes many years for children to develop the ability to efficiently apply spectral filters in selectively attending to a target (Lutfi et al., 2003; Leibold and Neff, 2011; Jones et al., 2015).

It is not yet entirely clear why, during decision-making, children exhibited looking behavior with eye gaze switching between target and non-target more often than adults. Auditory attention is still developing between 8 and 14 years of age among children (Klenberg et al., 2001). One possible explanation is that inattentiveness to the task might have manifested in frequent fixation switches. However, the effect of inattentiveness is considered small and does not contribute to any age difference in perceptual outcomes (see summary in Werner and Rubel, 1992). Our finding of similar ITD JNDs between children and adults also confirms that inattentiveness during task, if any, has very little impact on the perceptual outcome. Furthermore, frequent eye gaze switches observed in young children do not appear to be irrational due to inattention. This is because both adults and 11- to 14-year-old children showed more frequent fixation switches as ITD magnitude became smaller (Figure 5). Instead, the increased frequent fixation switches might be from strategies that children and adults used in coping with greater uncertainty during the decision-making process, especially when the saliency of the ITD cue is reduced.

Since children have demonstrated benefit from additional visual cues on tasks such as speech recognition (Wightman and Kistler, 2005) and working memory (Pillai and Yathiraj, 2017), we consider the possibility that, by looking to both the target and non-target, they were seeking additional visual cues to aid decision-making. One potential explanation stemming from these findings is that children may always seek additional visual cues to complement auditory cues during decision-making. However, the video sequence (Figure 1) provided no visual cues to accompany the direction of the target auditory stimulus, a fact that was conveyed *via* verbal instruction to the children during the task training process. Hence, it is unlikely that frequent fixation switching was a strategy that children adopted to gain additional visual information for the facilitation of left–right judgement. An alternative explanation is that listeners might go through a rehearsal phase during decision-making, by using fixations to the target and non-target to reinforce the internal category and compared it to the auditory target. When the auditory cue was salient enough for a listener to immediately sort it into a distinct category (i.e., left vs. right hemi-field), no comparison was necessary, hence reducing the likelihood of a fixation switch. However, when the ITD cue became less salient, listeners were more likely to seek a second perceptual “look” at the auditory target, by repeatedly rehearsing the internal mapping of ITD and comparing it with the small ITD cue to find the precise intracranial position. This would also explain in Figure 5 why some adults had more fixation switches when tested with small ITDs below 80 μs than with large ITDs. There is evidence to suggest that, in peripheral regions where ITD sensitivity is lower than that closer to mid-line (i.e., azimuth 0°), listeners can improve ITD JND by directing eye gaze to the target region (Maddox et al., 2014). Hence, we speculate that fixation switches might be a strategy to sharpen auditory sensitivity for less salient ITD cues during the rehearsal phase.

Finally, although ITD processing appears to be mature among children between 8 and 10 years old, real-time eye gaze suggests that children up to 14 years old may still be developing strategies to efficiently establish an internal mapping of intracranial positions in an ITD discrimination task. Developing executive functions, such as auditory working memory and attention, would also likely affect children’s ability to retain such an intracranial map of ITD magnitudes leading to increased fixation switches. Indeed, future work is needed to confirm and further address the mechanism underlying such a strategy in processing binaural cues.

## Summary and Conclusion

The present study examined the development of decision-making through eye gaze fixations during ITD processing. We modified a “looking-while-listening” paradigm to capture the time course of eye gaze fixations in a left–right discrimination task. The same task also measured listeners’ ITD JND thresholds. Adults and children had ITD JNDs that were consistent with previous findings (Goupell et al., 2013; Ehlers et al., 2016), which provided validation that the eye gaze paradigm accurately measured ITD sensitivity while capturing the decision-making process involved in discriminating sounds to the right vs. left, in both children and adults.

The finding in this study suggested that children between 8 and 14 years old typically took the same amount of time as an adult to make a right–left judgement, but appeared to show greater uncertainty prior to making their decision. The uncertainty in children was exhibited by fixation switches during the decision-making process, which might stem from a strategy invoked to sharpen sensitivity through eye gaze during a rehearsal phase after receiving the auditory input. As such, the age effect observed in fixation switches during ITD processing was dependent on the magnitude of the binaural cue. In conclusion, while children demonstrate adult-like sensitivity to ITD cues, findings from eye gaze behaviors revealed a processing strategy specific to uncertainty in decision-making that is still developing between the ages of 8 and 14 years.

## Data Availabiilty Statement

The datasets generated for this study are available on request to the corresponding author.

## Ethics Statement

All experimental protocols and procedures were reviewed and approved by the Health Sciences Institutional Review Board at the University of Wisconsin-Madison. All adult participants in the study provided written consent. For all child participants, parents or legal guardians signed informed consent and the children signed an assent form.

## Author Contributions

ZP and RL conceived and designed the study. ZP recruited participants, collected data, performed data analysis and interpretation, and wrote the manuscript. AK and RL provided input on results interpretation and revised the manuscript.

## Conflict of Interest

The authors declare that the research was conducted in the absence of any commercial or financial relationships that could be construed as a potential conflict of interest.
